# Ethyl 4-acetyl-5-oxo-3-phenyl­hexa­noate

**DOI:** 10.1107/S160053681100955X

**Published:** 2011-03-19

**Authors:** Hongwei Wang, Yimin Hu

**Affiliations:** a College of Chemistry and Materials Science, Anhui Key Laboratory of Molecular-Based Materials, Anhui Normal University, Wuhu, Anhui 241000, People’s Republic of China

## Abstract

The reaction of ethyl 3-bromo-3-phenyl­propano­ate with pentane-2,4-dione, in the presence of palladium(II) acetate and triphenyl­phosphine, in dimethyl­formamide, unexpectedly gave the title product, C_16_H_20_O_4_. The mol­ecule contains one chiral C atom but the crystal is racemic. In the crystal, neighboring mol­ecules form a chain along [100] through three weak C—H⋯O inter­actions. Furthermore, a double-stranded structure is formed through weak C—H⋯O inter­actions between two parallel chains.

## Related literature

For Pd-catalysed coupling reactions, see: Hu *et al.* (2008 ▶); Hu, Ouyang *et al.* (2009 ▶); Hu, Yu *et al.* (2009 ▶). For the biological activity of pentane-2,4-dione derivatives, see: Vijaikumar & Pitchumani (2010 ▶). For related structures, see: Hu, Lin *et al.* (2010 ▶); Hu, Ren *et al.* (2010 ▶).
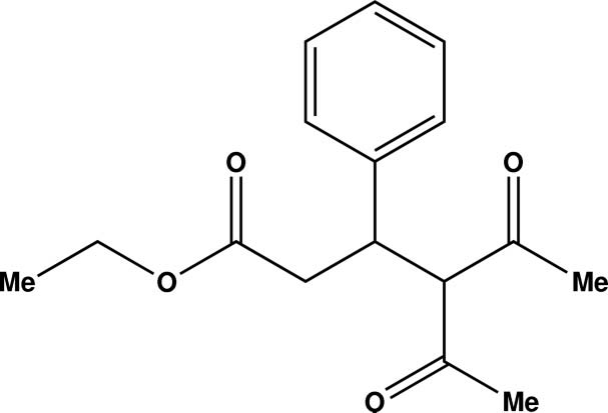


## Experimental

### 

#### Crystal data

C_16_H_20_O_4_
*M* *_r_* = 276.32
Triclinic, 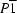

*a* = 5.8213 (11) Å
*b* = 7.7638 (18) Å
*c* = 17.8532 (15) Å
α = 80.973 (2)°
β = 88.977 (3)°
γ = 75.033 (2)°
*V* = 769.6 (2) Å^3^
*Z* = 2
Mo *K*α radiation
μ = 0.09 mm^−1^
*T* = 291 K
0.28 × 0.24 × 0.22 mm

#### Data collection

Bruker SMART APEX CCD diffractometer
Absorption correction: multi-scan (*SADABS*; Bruker, 2000 ▶) *T*_min_ = 0.977, *T*_max_ = 0.982
8564 measured reflections
3033 independent reflections
1726 reflections with *I* > 2σ(*I*)
*R*_int_ = 0.050

#### Refinement

*R*[*F* ^2^ > 2σ(*F* ^2^)] = 0.050
*wR*(*F* ^2^) = 0.099
*S* = 1.07
3033 reflections
185 parameters
H-atom parameters constrained
Δρ_max_ = 0.16 e Å^−3^
Δρ_min_ = −0.15 e Å^−3^

### 

Data collection: *SMART* (Bruker, 2000 ▶); cell refinement: *SAINT* (Bruker, 2000 ▶); data reduction: *SAINT*; program(s) used to solve structure: *SHELXTL* (Sheldrick, 2008 ▶); program(s) used to refine structure: *SHELXTL*; molecular graphics: *SHELXTL*; software used to prepare material for publication: *SHELXTL*.

## Supplementary Material

Crystal structure: contains datablocks global, I. DOI: 10.1107/S160053681100955X/bh2342sup1.cif

Structure factors: contains datablocks I. DOI: 10.1107/S160053681100955X/bh2342Isup2.hkl

Additional supplementary materials:  crystallographic information; 3D view; checkCIF report

## Figures and Tables

**Table 1:** Hydrogen-bond geometry (Å, °)

*D*—H⋯*A*	*D*—H	H⋯*A*	*D*⋯*A*	*D*—H⋯*A*
C6—H6⋯O1^i^	0.93	2.63	3.534 (2)	165
C8—H8*B*⋯O1^i^	0.97	2.70	3.525 (2)	144
C12—H12⋯O1^i^	0.98	2.46	3.387 (2)	157
C14—H14*C*⋯O4^ii^	0.96	2.72	3.405 (2)	129

Symmetry codes: (i) 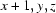; (ii) 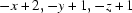.

**Fig. 1.**
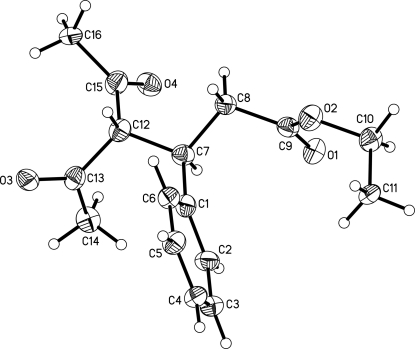
A view of the title compound showing displacement ellipsoids drawn at 30% probability level.

**Fig. 2.**
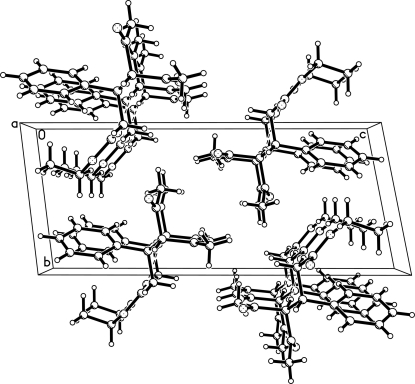
A view of the crystal packing of the title compound down a axis.

**Fig. 3.**
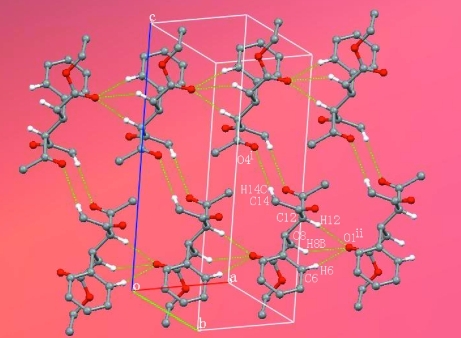
A view of the double-stranded structure (i: 2-x, 1-y, 1-z; ii: 1+x, y, z).

**Fig. 4.**
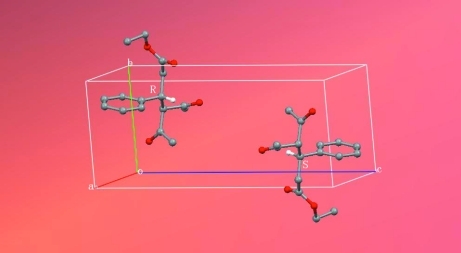
A view of a pair of recemic molecules.

## supplementary crystallographic information

### Comment

Palladium-catalyzed coupling reactions have become an important tool in modern organic synthesis chemistry (Hu *et al.* 2008). They have made a wide variety of active pharmaceutical ingredients, natural substances and other complex organic molecules economically accessible (Hu & Yu *et al.*, 2009; Hu, Ouyang *et al.*, 2009). The pentane-2,4-dione derivatives, which have physiological activity, are effective intermediates in the synthesis of many complex natural products (Vijaikumar & Pitchumani, 2010). We have reported some novel palladium-catalyzed intermolecular and intramolecular reactions of aryl halides with the olefins and diynes (Hu, Lin *et al.*, 2010; Hu, Ren *et al.*, 2010). The reaction of ethyl 3-bromo-3-phenylpropanoate with pentane-2,4-dione, in the presence of palladium(II) acetate and triphenylphosphine, in DMF at 373 K for 22 h, gave the unexpected title product.

The molecular structure of the title compound, C_16_H_20_O_4_, reveals that all the bond lengths and angles have normal values. As shown in Fig. 1, one chiral carbon, C7, was observed in the molecule. Due to the existence of inversion centers in the crystal packing, the C7 atoms exhibit *R*-conformation in the half of the molecules, and display *S*-conformation in the other half of the molecules. So the whole crystal is racemic (Fig. 4). In the crystal packing, the weak C—H···O interactions play important roles. Neighboring molecules form a one dimensional chain through the weak C6—H6···O1^ii^, C8—H8b···O1^ii^ and C12—H12···O1^ii^ (ii: 1+*x*, *y*, *z*) interactions (Fig. 2). Furthermore, two neighboring chains are parallel to each other to form a double-stranded structure through the weak C14—H14C···O4^i^ (i: 2-*x*, 1-*y*, 1-*z*) interactions (Fig. 3).

### Experimental

An oven-dried Schlenk flask was evacuated, filled with nitrogen, and then charged with pentane-2,4-dione (1.00 g, 10 mmol), ethyl-3-bromo-3-phenylpropanoate (2.82 g, 11 mmol), tributylamine (3 ml), PPh_3_ (52.5 mg, 0.2 mmol), Pd(OAc)_2_ (24 mg, 0.1 mmol), and DMF (10 ml) to give a yellow solution. The reaction mixture was heated at 373 K with stirring. The reaction mixture was cooled to room temperature after 22 h and the resulting yellow-orange mixture was diluted with Et_2_O (10 ml). The mixture was washed with H_2_O (15 ml) and the aqueous layer was extracted with Et_2_O (20 ml). The combined organic layers were dried (MgSO_4_), filtered, and concentrated *in vacuo*. The crude material was purified by flash column chromatography on silica gel (petroleum ether:EtOAc, 9:1) and recrystallized from EtOAc, yield 2.27 g (82%). Colorless crystals suitable for X-ray diffraction were obtained by recrystallization from a solution of the title compound from ethyl acetate, over a period of one week.

### Refinement

H atoms were positioned geometrically and refined using a riding model (including free rotation about the methyl C—C bond), with C—H = 0.93–0.97 Å and with *U*_iso_(H) = 1.2 (1.5 for methyl groups) times *U*_eq_(carrier C).

### Crystal data

**Table:**

C_16_H_20_O_4_	*Z* = 2
*M**_r_* = 276.32	*F*(000) = 296
Triclinic, *P*1	*D*_x_ = 1.192 Mg m^−^^3^
Hall symbol: -P 1	Mo *K*α radiation, λ = 0.71073 Å
*a* = 5.8213 (11) Å	Cell parameters from 3519 reflections
*b* = 7.7638 (18) Å	θ = 2.2–23.2°
*c* = 17.8532 (15) Å	µ = 0.09 mm^−^^1^
α = 80.973 (2)°	*T* = 291 K
β = 88.977 (3)°	Block, colourless
γ = 75.033 (2)°	0.28 × 0.24 × 0.22 mm
*V* = 769.6 (2) Å^3^	

### Data collection

**Table:**

Bruker SMART APEX CCD diffractometer	3033 independent reflections
Radiation source: sealed tube	1726 reflections with *I* > 2σ(*I*)
graphite	*R*_int_ = 0.050
φ and ω scans	θ_max_ = 26.0°, θ_min_ = 1.2°
Absorption correction: multi-scan (*SADABS*; Bruker, 2000)	*h* = −7→7
*T*_min_ = 0.977, *T*_max_ = 0.982	*k* = −9→9
8564 measured reflections	*l* = −21→21

### Refinement

**Table:**

Refinement on *F*^2^	Secondary atom site location: difference Fourier map
Least-squares matrix: full	Hydrogen site location: inferred from neighbouring sites
*R*[*F*^2^ > 2σ(*F*^2^)] = 0.050	H-atom parameters constrained
*wR*(*F*^2^) = 0.099	*w* = 1/[σ^2^(*F*_o_^2^) + (0.03*P*)^2^] where *P* = (*F*_o_^2^ + 2*F*_c_^2^)/3
*S* = 1.07	(Δ/σ)_max_ < 0.001
3033 reflections	Δρ_max_ = 0.16 e Å^−^^3^
185 parameters	Δρ_min_ = −0.15 e Å^−^^3^
0 restraints	Extinction correction: *SHELXTL* (Sheldrick, 2008), Fc^*^=kFc[1+0.001xFc^2^λ^3^/sin(2θ)]^-1/4^
0 constraints	Extinction coefficient: 0.017 (3)
Primary atom site location: structure-invariant direct methods	

### Fractional atomic coordinates and isotropic or equivalent isotropic displacement parameters (Å^2^)

**Table:**

	*x*	*y*	*z*	*U*_iso_*/*U*_eq_	
C1	0.9743 (3)	0.8135 (3)	0.22191 (11)	0.0424 (5)	
C2	0.8295 (4)	0.7260 (3)	0.19182 (11)	0.0473 (5)	
H2	0.7199	0.6823	0.2226	0.057*	
C3	0.8438 (4)	0.7019 (3)	0.11675 (11)	0.0463 (5)	
H3	0.7450	0.6411	0.0978	0.056*	
C4	1.0006 (4)	0.7658 (3)	0.07023 (12)	0.0464 (5)	
H4	1.0075	0.7507	0.0195	0.056*	
C5	1.1474 (3)	0.8521 (3)	0.09850 (11)	0.0463 (5)	
H5	1.2557	0.8957	0.0671	0.056*	
C6	1.1361 (3)	0.8750 (3)	0.17330 (11)	0.0436 (5)	
H6	1.2389	0.9329	0.1920	0.052*	
C7	0.9534 (4)	0.8488 (3)	0.30314 (11)	0.0411 (5)	
H7	0.8304	0.7949	0.3272	0.049*	
C8	0.8736 (3)	1.0549 (3)	0.30396 (11)	0.0400 (5)	
H8A	0.8589	1.0774	0.3560	0.048*	
H8B	0.9929	1.1111	0.2802	0.048*	
C9	0.6410 (3)	1.1362 (3)	0.26269 (11)	0.0411 (5)	
C10	0.4381 (4)	1.3543 (3)	0.16176 (11)	0.0488 (5)	
H10A	0.4133	1.4844	0.1540	0.059*	
H10B	0.3032	1.3256	0.1887	0.059*	
C11	0.4545 (4)	1.2916 (3)	0.08822 (11)	0.0470 (5)	
H11A	0.5764	1.3323	0.0593	0.070*	
H11B	0.3048	1.3394	0.0612	0.070*	
H11C	0.4931	1.1621	0.0960	0.070*	
C12	1.1855 (3)	0.7662 (3)	0.34946 (11)	0.0409 (5)	
H12	1.3046	0.8279	0.3281	0.049*	
C13	1.2830 (3)	0.5652 (3)	0.34769 (11)	0.0441 (5)	
C14	1.1213 (4)	0.4433 (3)	0.36507 (12)	0.0490 (5)	
H14A	1.2115	0.3200	0.3675	0.074*	
H14B	1.0003	0.4717	0.3259	0.074*	
H14C	1.0483	0.4599	0.4130	0.074*	
C15	1.1577 (4)	0.7879 (3)	0.43302 (12)	0.0498 (5)	
C16	1.3691 (4)	0.8014 (3)	0.47412 (12)	0.0471 (5)	
H16A	1.3837	0.9232	0.4638	0.071*	
H16B	1.5089	0.7214	0.4577	0.071*	
H16C	1.3520	0.7683	0.5276	0.071*	
O1	0.4638 (2)	1.0866 (2)	0.27737 (8)	0.0483 (4)	
O2	0.6542 (2)	1.2691 (2)	0.20664 (8)	0.0509 (4)	
O3	1.4918 (2)	0.50694 (19)	0.33553 (7)	0.0458 (4)	
O4	0.9718 (2)	0.7909 (2)	0.46421 (8)	0.0488 (4)	

### Atomic displacement parameters (Å^2^)

**Table:**

	*U*^11^	*U*^22^	*U*^33^	*U*^12^	*U*^13^	*U*^23^
C1	0.0433 (11)	0.0391 (11)	0.0406 (10)	−0.0055 (9)	−0.0051 (8)	−0.0021 (8)
C2	0.0545 (12)	0.0471 (13)	0.0438 (11)	−0.0141 (10)	−0.0010 (9)	−0.0156 (9)
C3	0.0473 (12)	0.0497 (12)	0.0450 (11)	−0.0135 (10)	−0.0033 (9)	−0.0146 (9)
C4	0.0454 (11)	0.0485 (12)	0.0480 (12)	−0.0147 (10)	−0.0068 (9)	−0.0103 (9)
C5	0.0433 (12)	0.0503 (13)	0.0472 (12)	−0.0173 (10)	0.0016 (9)	−0.0047 (9)
C6	0.0412 (11)	0.0426 (12)	0.0476 (12)	−0.0110 (9)	−0.0007 (9)	−0.0080 (9)
C7	0.0464 (11)	0.0380 (11)	0.0403 (11)	−0.0132 (9)	0.0007 (8)	−0.0061 (8)
C8	0.0374 (10)	0.0419 (11)	0.0428 (11)	−0.0108 (9)	0.0021 (8)	−0.0122 (9)
C9	0.0432 (12)	0.0350 (11)	0.0471 (11)	−0.0120 (9)	0.0015 (9)	−0.0095 (8)
C10	0.0559 (13)	0.0427 (12)	0.0440 (11)	−0.0128 (10)	−0.0086 (9)	0.0062 (9)
C11	0.0435 (11)	0.0469 (12)	0.0532 (12)	−0.0131 (9)	−0.0146 (9)	−0.0118 (10)
C12	0.0341 (10)	0.0470 (12)	0.0445 (11)	−0.0182 (9)	0.0047 (8)	−0.0034 (9)
C13	0.0344 (11)	0.0526 (12)	0.0452 (11)	−0.0115 (9)	0.0020 (8)	−0.0066 (9)
C14	0.0525 (13)	0.0453 (13)	0.0474 (12)	−0.0148 (10)	0.0069 (9)	0.0013 (10)
C15	0.0417 (12)	0.0572 (14)	0.0509 (12)	−0.0127 (10)	−0.0015 (10)	−0.0095 (10)
C16	0.0522 (12)	0.0447 (11)	0.0487 (11)	−0.0125 (10)	−0.0114 (9)	−0.0183 (9)
O1	0.0402 (8)	0.0558 (9)	0.0467 (8)	−0.0135 (7)	−0.0032 (6)	0.0010 (7)
O2	0.0538 (9)	0.0545 (9)	0.0414 (8)	−0.0152 (7)	−0.0036 (6)	0.0040 (7)
O3	0.0435 (8)	0.0476 (9)	0.0474 (8)	−0.0082 (6)	0.0065 (6)	−0.0177 (6)
O4	0.0493 (9)	0.0499 (9)	0.0495 (8)	−0.0138 (7)	0.0131 (7)	−0.0142 (7)

### Geometric parameters (Å, °)

**Table:**

C1—C2	1.374 (3)	C10—O2	1.450 (2)
C1—C6	1.393 (3)	C10—C11	1.464 (3)
C1—C7	1.515 (3)	C10—H10A	0.9700
C2—C3	1.380 (3)	C10—H10B	0.9700
C2—H2	0.9300	C11—H11A	0.9600
C3—C4	1.359 (3)	C11—H11B	0.9600
C3—H3	0.9300	C11—H11C	0.9600
C4—C5	1.360 (3)	C12—C13	1.522 (3)
C4—H4	0.9300	C12—C15	1.528 (3)
C5—C6	1.373 (3)	C12—H12	0.9800
C5—H5	0.9300	C13—O3	1.211 (2)
C6—H6	0.9300	C13—C14	1.495 (3)
C7—C12	1.532 (3)	C14—H14A	0.9600
C7—C8	1.549 (3)	C14—H14B	0.9600
C7—H7	0.9800	C14—H14C	0.9600
C8—C9	1.491 (3)	C15—O4	1.205 (2)
C8—H8A	0.9700	C15—C16	1.479 (3)
C8—H8B	0.9700	C16—H16A	0.9600
C9—O1	1.202 (2)	C16—H16B	0.9600
C9—O2	1.337 (2)	C16—H16C	0.9600
			
C2—C1—C6	116.86 (19)	C11—C10—H10A	109.6
C2—C1—C7	121.89 (19)	O2—C10—H10B	109.6
C6—C1—C7	121.21 (19)	C11—C10—H10B	109.6
C1—C2—C3	121.2 (2)	H10A—C10—H10B	108.1
C1—C2—H2	119.4	C10—C11—H11A	109.5
C3—C2—H2	119.4	C10—C11—H11B	109.5
C4—C3—C2	120.8 (2)	H11A—C11—H11B	109.5
C4—C3—H3	119.6	C10—C11—H11C	109.5
C2—C3—H3	119.6	H11A—C11—H11C	109.5
C3—C4—C5	119.4 (2)	H11B—C11—H11C	109.5
C3—C4—H4	120.3	C13—C12—C15	106.57 (16)
C5—C4—H4	120.3	C13—C12—C7	113.03 (16)
C4—C5—C6	120.2 (2)	C15—C12—C7	112.55 (16)
C4—C5—H5	119.9	C13—C12—H12	108.2
C6—C5—H5	119.9	C15—C12—H12	108.2
C5—C6—C1	121.58 (19)	C7—C12—H12	108.2
C5—C6—H6	119.2	O3—C13—C14	121.6 (2)
C1—C6—H6	119.2	O3—C13—C12	119.19 (18)
C1—C7—C12	112.74 (16)	C14—C13—C12	119.09 (17)
C1—C7—C8	109.58 (15)	C13—C14—H14A	109.5
C12—C7—C8	109.89 (15)	C13—C14—H14B	109.5
C1—C7—H7	108.2	H14A—C14—H14B	109.5
C12—C7—H7	108.2	C13—C14—H14C	109.5
C8—C7—H7	108.2	H14A—C14—H14C	109.5
C9—C8—C7	110.65 (15)	H14B—C14—H14C	109.5
C9—C8—H8A	109.5	O4—C15—C16	121.6 (2)
C7—C8—H8A	109.5	O4—C15—C12	121.10 (18)
C9—C8—H8B	109.5	C16—C15—C12	117.33 (19)
C7—C8—H8B	109.5	C15—C16—H16A	109.5
H8A—C8—H8B	108.1	C15—C16—H16B	109.5
O1—C9—O2	124.06 (18)	H16A—C16—H16B	109.5
O1—C9—C8	124.11 (18)	C15—C16—H16C	109.5
O2—C9—C8	111.82 (16)	H16A—C16—H16C	109.5
O2—C10—C11	110.33 (17)	H16B—C16—H16C	109.5
O2—C10—H10A	109.6	C9—O2—C10	116.13 (15)
			
C6—C1—C2—C3	−0.4 (3)	C1—C7—C12—C13	−54.2 (2)
C7—C1—C2—C3	177.12 (18)	C8—C7—C12—C13	−176.77 (16)
C1—C2—C3—C4	−0.7 (3)	C1—C7—C12—C15	−175.02 (18)
C2—C3—C4—C5	1.0 (3)	C8—C7—C12—C15	62.4 (2)
C3—C4—C5—C6	−0.3 (3)	C15—C12—C13—O3	−102.9 (2)
C4—C5—C6—C1	−0.8 (3)	C7—C12—C13—O3	132.96 (18)
C2—C1—C6—C5	1.1 (3)	C15—C12—C13—C14	73.9 (2)
C7—C1—C6—C5	−176.43 (18)	C7—C12—C13—C14	−50.2 (2)
C2—C1—C7—C12	122.1 (2)	C13—C12—C15—O4	−93.4 (2)
C6—C1—C7—C12	−60.5 (2)	C7—C12—C15—O4	31.0 (3)
C2—C1—C7—C8	−115.2 (2)	C13—C12—C15—C16	85.4 (2)
C6—C1—C7—C8	62.2 (2)	C7—C12—C15—C16	−150.14 (18)
C1—C7—C8—C9	58.7 (2)	O1—C9—O2—C10	−0.5 (3)
C12—C7—C8—C9	−176.91 (16)	C8—C9—O2—C10	178.60 (17)
C7—C8—C9—O1	55.4 (3)	C11—C10—O2—C9	−103.9 (2)
C7—C8—C9—O2	−123.67 (17)		

### Hydrogen-bond geometry (Å, °)

**Table:**

*D*—H···*A*	*D*—H	H···*A*	*D*···*A*	*D*—H···*A*
C6—H6···O1^i^	0.93	2.63	3.534 (2)	165
C8—H8B···O1^i^	0.97	2.70	3.525 (2)	144
C12—H12···O1^i^	0.98	2.46	3.387 (2)	157
C14—H14C···O4^ii^	0.96	2.72	3.405 (2)	129

Symmetry codes: (i) *x*+1, *y*, *z*; (ii) −*x*+2, −*y*+1, −*z*+1.
